# Detecting the neuropathic pain component in the clinical setting: a study protocol for validation of screening instruments for the presence of a neuropathic pain component

**DOI:** 10.1186/1471-2377-14-94

**Published:** 2014-05-02

**Authors:** Hans Timmerman, Oliver Wilder-Smith, Chris van Weel, André Wolff, Kris Vissers

**Affiliations:** 1 Department of Anesthesiology, Pain and Palliative Medicine, Radboud University medical center, Huispost 717, PO Box 9101, 6500 HB Nijmegen, the Netherlands; 2 Department of Primary and Community Care, Radboud University medical center, Nijmegen, the Netherlands; 3 Australian Primary Health Care Research Institute, Australian National University, Canberra, Australia

**Keywords:** PainDETECT questionnaire, PDQ, DN4, Validation, Low back pain, Neck-shoulder pain, Peripheral nerve damage

## Abstract

**Background:**

The presence of nerve damage plays a key role in the development and prognosis of chronic pain states. Assessment of the presence and severity of a neuropathic pain component (NePC) is key in diagnosing chronic pain patients. Low back pain (LBP) and neck and shoulder pain (NSP) are highly prevalent and clinically important medical and societal problems in which a NePC is frequently present. The more severe the NePC, the worse the course of the pain, its prognosis and the results of treatment. Reliable and standardised diagnosis of the NePC remains difficult to achieve. Standardized and validated screening tools may help to reliably identify the NePC in individual chronic pain patients. The aim of this study is to validate the Dutch language versions of the Pain*DETECT* Questionnaire (PDQ_-Dlv_) and the ‘Douleur Neuropathique 4 Questions’ (DN4_-Dlv_) for use in primary and specialist medical care settings to screen for a NePC in patients with chronic pain due to (1) LBP, (2) NSP or (3) known peripheral nerve damage (PND).

**Methods/design:**

The study design is cross-sectional to assess the validity of the PDQ_-Dlv_ and the DN4_-Dlv_ with 2 weeks follow-up for test-retest reliability and 3 months follow-up for monitoring and prognosis. 438 patients with chronic pain due to (1) LBP, (2) NSP or (3) PND. will be included in this study. Based on the IASP definition of neuropathic pain, two physicians will independently assess whether the patient has a NEPC or not. This result will be compared with the outcome of the PDQ_-Dlv_ & DN4_-Dlv_, the grading system for neuropathic pain, bed side examination and quantitative sensory testing. This study will further collect data regarding prevalence of NePC, general health status, mental health status, functioning, pain attribution and quality of life.

**Discussion:**

The rationale for this study is to provide detailed information on the clinimetric quality of the PDQ_-Dlv_ and DN4_-Dlv_ in Dutch speaking countries. Our innovative multi-factorial approach should help achieve more reliable diagnosis and quantification of a NePC in patients with chronic pain.

**Trial registration:**

The Netherlands National Trial Register (NTR3030).

## Background

The International Association for the Study of Pain (IASP, 2011) defines Neuropathic Pain (NeP) as ‘pain caused by a lesion or disease of the somatosensory nervous system’ (http://www.iasp-pain.org/Education/Content.aspx?ItemNumber=1698#Neuropathicpain). This definition will be used in this study because of its diagnostic specificity, anatomic precision and the usefulness in clinical as well as research conditions [1]. NeP plays an important role in the development and prognosis of chronic pain states. A relevant example is patients with low back pain (LBP) and neck-shoulder pain (NSP), which are both highly prevalent and clinically important medical and societal problems: In this context, the more severe the NeP, the worse the pain course, the prognosis and the results of treatment [2-5]*.*

**Figure 1:**
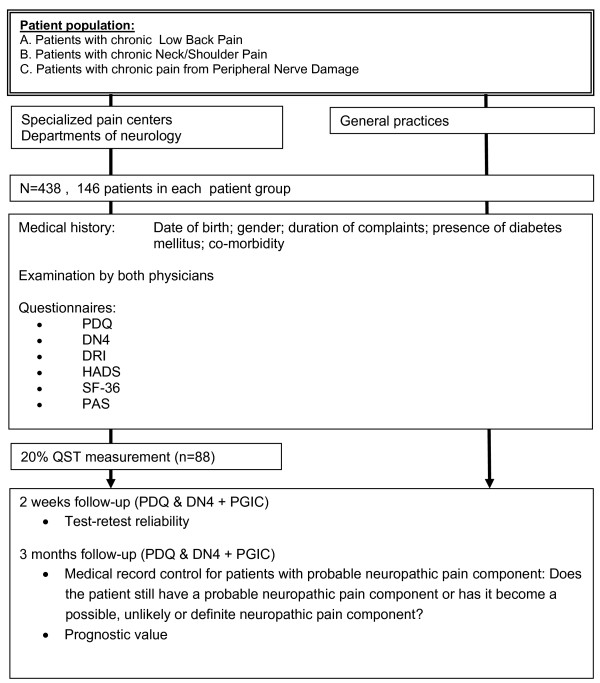
**Flow-diagram of the study.** PDQ: Pain*DETECT* questionnaire; DN4: Douleur neuropatique 4 questions; DRI : Disability rating index; HADS: Hospital anxiety depression scale; RAND-36 : RAND 36-item health survey; PAS: Pain attribution scale; QST: Quantitative sensory testing; PGIC: Patients global impression of change.

The incidence of NeP in the Dutch general population is 0.81% or 130.000 new patients in the Netherlands per year. NeP is 63% more common in women than in men and peaks between 70 and 79 years of age [6]. LBP as well as NSP are among the top 10 health problems encountered in general practice. For men and women, respectively, the prevalence of LBP and/or NSP in the general practice is in the range of 55 – 86 and 24 - 113 per 1000 patients a year. In general practice, radiating pain from the low back or neck occurs in men and women in respectively 4 - 8 and 10 patients per 1000 patients [7]/(http://www.nationaalkompas.nl/gezondheid-en-ziekte/ziekten-en-aandoeningen/bewegingsstelsel-en-bindweefsel/nek-en-rugklachten/omvang/). The prevalence of chronic pain syndromes due to peripheral nerve damage (PND) is 3,3 per 1000 per year [8].

Strictly speaking, the diagnosis of neuropathic pain is a patho-anatomical diagnosis presuming knowledge regarding nerve injury which is difficult to obtain in the clinical situation. Thus in the clinical context it is better to speak of a neuropathic pain component (NePC), which is a clinical syndrome based on a typical set of clinical symptoms and signs. Clinically, a NePC is characterized by spontaneous pain and abnormal pain sensations [9]. NeP is typically described as a spontaneous ongoing burning or shooting pain with spontaneous sharp exacerbations and somatosensory abnormalities after a (non-) noxious stimulus [10].

As a rule, a NePC has a considerable impact on the quality of daily life [6]. Hence it is important for physicians in daily practice (specialist care as well as primay care) to diagnose the presence and severity of a NePC in individual patients. In clinical practice it is, however, often difficult to reliably diagnose a NePC in (sub)acute and chronic pain of the low back and neck shoulder region. The diagnosis of a NePC is at present primarily based on clinical examination by a physician including sensory examination. Quantitative sensory testing (QST) may provide extra information for selected clinical cases and in the research context [11,12].

Because a reliable diagnosis of the neuropathic pain component is often difficult to accomplish in routine practice [2], it would be helpful to have a screening tool to detect such a component for clinical triage and epidemiological purposes [12,13]. Apart from optimal sensitivity and specificity, such a screening tool should be easy to use in clinical practice, not only for the first visit but also during follow up. The availability of such a simple, validated, Dutch language screening tool should improve diagnosis and quantification of a NePC and hence lead to better therapy. At present, no specific (validated) instrument to determine the neuropathic component in LBP, NSP and PND is available in the Dutch language. The Pain*DETECT*-Questionnaire (PDQ) [2] and the Douleur Neuropatique 4 Questions (DN4) [14] were originally developed and validated in Germany and France, respectively. Both are considered to be reliable screening tools with a high sensitivity, specificity and positive predictive value. Recently, the DN4 and the PDQ have been translated into a Dutch language version (Dlv) by Van Seventer et al [15] and Timmerman et al [16]*,* respectively.

Validation of the Dutch versions of DN4_-Dlv_ and PDQ_-Dlv_ will improve the identification of a NePC in Dutch primary and specialist medical care, also facilitating remote follow up evaluation by telephone, internet or post for clinical and scientific purposes. We chose an innovative approach which should lead to a more reliable identification and quantification of a NePC in patients with chronic pain. This study will help define patient groups at risk for a NePC and will help to understand and assess the variability and burden of a NePC in individual patients.

The aim of this study is to establish the clinimetric quality, including 2-weeks test-retest reliability, of the PDQ_-Dlv_ and the DN4_-Dlv_ for use in primary care and specialist medical care settings in Dutch speaking countries for patients with chronic pain due to LBP, NSP or known peripheral nerve damage (PND). Follow-up for monitoring and prognosis properties of DN4_-Dlv_ and PDQ_-Dlv_ for a NePC will be done over a period of 3 months. Additional data will be collected regarding NEPC prevalence, general health status, mental health status, functioning, pain attribution and health related quality of life in patients with chronic pain.

## Methods

The medical and ethical review board Committee on Research Involving Human Subjects region Arnhem-Nijmegen, Nijmegen, the Netherlands, has given approval to conduct this study, Dossier number: 2008/348; NL 25343.091.08; The Netherlands National Trial Register NTR3030.

## Design

In this study a cross-sectional, observational, research design with 3-months follow up will be used to study the clinimetric quality of the DN4_-Dlv_ and PDQ_-Dlv_ (Figure 1).

### Setting

Multicenter recruitment will be take place in academic pain centres, non-academic pain centres and non-academic departments of neurology. Patients will be seen by the two physicians during normal office hours, or when that is not possible during a special office hour for this study. Furthermore, patients willing to participate in this study from general practices will also be included in this study via a special office hour in the clinical trainings centre of Radboud university medical center (Radboudumc). Each patient will be seen by two physicians, independently of each other, working in the same institute. The medical background of the participating physicians is diverse (experienced pain specialists, pain specialist trainees, experienced neurologists and experienced general practitioners).

### Participants

The patients will be recruited non-selectively and consecutively in the period from September 2009 till July 2013. *Inclusion criteria:* Male and female adult patients (>18 years of age) with chronic (>3 months) LBP or NSP radiating into respectively leg(s) or arm(s) or patients with chronic pain due to PND. *Exclusion criteria:* Patients diagnosed with malignancy, compression fractures, patients with painful syndromes of unknown origin or associated with diffuse pains (such as ankylosing spondylitis or fibromyalgia), severe mental illness, chronic alcoholism or substance abuse, inability to fill in the questionnaire adequately, or incapable of understanding Dutch. Subjects can leave the study at any time for any reason without any consequences. The investigator can decide to withdraw a subject from the study for urgent medical reasons. When an individual subject leaves the study all the information from the patient will be kept in the database, and possibly be used for data analysis or withdrawal analysis. Such patients will be replaced.

### Measurements

At the first visit, each patient will be seen by two physicians who will question and examine the patients. They will then independently classify the patients’ pain as pain with or without a NePC, based in the IASP NeP criteria and supported by a standardized assessment form. Next, the patient will complete seven questionnaires (including the PDQ and the DN4). Twenty percent of the patients will additionally undergo QST measurement following the first visit. Two weeks and three months after the initial visit three follow-up questionnaires will be sent to the patient by mail.

#### Demographics

Date of birth, gender, weight (Kg), length (m), nationality, nationality of origin, pain medication, smoking (cigarettes a day), alcohol consumption (units per day) and education level will be assessed by use of a self completed questionnaire. Pain at this moment will be assessed by use of a numeric rating scale (0-10, NRS) Medical co-morbidity, duration of complaints (years/months), presence of diabetes mellitus (yes/no), presence of HIV (yes/no), presence of herpes (yes/no) and undergoing of chemotherapy (yes/no) are based on interview by the physician and noted by the physician on the standardized assessment form.

#### Pain classification

At each centre participating patients will be examined by two (rater A and B) independent and trained pain physicians, two experienced neurologists or two experienced general practitioners, working independently of each other and blinded to the diagnosis of the other physician. To achieve standardization of the history and clinical examination all participating physicians will be trained at the sites. Both physicians will classify the pain regarding the presence or absence of a NePC based on history and clinical examination. Level of certainty of the physicians regarding the pain component classification will be assessed by use of a Visual Analogue Scale (VAS, range 0-100). The findings are noted on the standardized assessment form by the physician. To monitor the quality of the clinical examination random quality checks, by expected/ unexpected visits, will be used.

#### Grading system

The grading system for neuropathic pain as proposed by Treede et al. [1] will be used as a secondary comparison with the outcome of both the PDQ_-Dlv_ and DN4_-Dlv_ and with the outcome of the original pain classification by the two physicians. This system provides a working hypothesis for the origin of patients’ pain. The criteria are graded on basis of history and testing in medical examination [1]: (1) Pain with a distinct neuroanatomically plausible distribution; (2) A history suggestive of a relevant lesion or disease affecting the peripheral or central somatosensory system; (3) Demonstration of the distinct neuroanatomically plausible distribution by at least one confirmatory test; (4) Demonstration of the relevant lesion or disease by at least one confirmatory test lesion or disease explaining NeP. When the criteria 1-4 are all fulfilled the pain will be graded as possessing a ‘definite NePC’. Criteria 1 and 2 and either 3 or 4 will be graded as ‘probable NePC’. Criteria 1 & 2 without criteria 3 or 4 will be graded as ‘possible NePC’. The pain is ‘unlikely to have a NePC’ when no criteria, or only criteria 1 or 2, are graded as present (Table 1). The answers (yes or no) to the four criteria are noted by the physician on the standardized assessment form. Three months after the first consultation by the two physicians the medical record of patients with ‘probable NePC’ according to the grading system will be controlled: i.e. does the patient still have probable NePC, or does he now have definite NePC?.

**Table 1:** **Outcome of the grading system **[1]

***Criteria 1:***	***Criteria 2:***	***Criteria 3:***	***Criteria 4:***	***Outcome of the grading system***
**Pain with a distinct neuro-anatomically plausible distribution**	**History suggestive of a relevant lesion or disease affecting the peripheral or central somatosensory system**	**Demonstration of the distinct neuroanatomically plausible distribution by at least one confirmatory test**	**Demonstration of the relevant lesion or disease by at least one confirmatory test lesion or disease explaining NeP**	
*-*	*-*	*-*	*-*	***Unlikely to be NeP***
*V*	*-*	*-*	*-*	***Unlikely to be NeP***
*-*	*V*	*-*	*-*	***Unlikely to be NeP***
*V*	*V*	*-*	*-*	***Possible NeP***
*V*	*V*	*V*	*-*	***Probable NeP***
*V*	*V*	*-*	*V*	***Probable NeP***
*V*	*V*	*V*	*V*	***Definite NeP***

NeP: Neuropathic Pain; V: present; -: absent.

#### Bedside examination

Bed-side examination of the patient is performed by both physicians. The aim of this examination is to find possible abnormalities suggestive for a relevant lesion or disease which affects the peripheral or central somatosensory system [17]. The value of bed-side examination within the clinical examination is that it will give insight in the pathology and the localization of the lesion or disease which is causing the pain. Touch, pin prick, pressure, cold, heath, vibration and temporal summation were included in the examination to provide proof of a NePC [10,12,18]. This evaluation will be assessed in a standardized way. The location indicated by the patient as the one with maximum pain will be compared with the mirrored location on the contra lateral side. If the pain has a double sided character a location without pain as close as possible to the original mirror site will be tested for comparison. The outcome is noted by the physician on the standardized assessment form: a) Is there a sensation b) is the sensation unpleasant or c) is the sensation painful (all scored as Yes, No or Unclear). The response of the patient will be noted on the assessment form. The following tests will be performed in all patients: *Mechanical static allodynia * by blunt pressure with a finger at a force which normally doesn’t evoke pain; *Dynamic mechanical allodynia* by stroking the skin with a Soft Brush (SENSElab^TM^, Brush-05, Somedic AB, Hörby, Sweden), one movement of 1-2 centimeter and three movements of 1-2 centimeters (wind-up response); *Mechanical pinprick allodynia* by touch of the skin with a plastic safety pin and a Von Frey hair (TOUCH TEST^R^, 5.07, 10.0 g, North Coast Medical Inc., Gilroy, USA). *Heat allodynia* by using TipTherm^R^ (TipTherm, Brüggen, Germany) in a baby-bottle warmer (ISI mini Baby Bottle Warmer, Assen, the Netherlands) set at 45 degrees Celsius; *Cold allodynia* with an ice cube placed on the skin for 2 seconds and *Vibration* with a Tuning fork (128 Hz; Medipharchem, Wormerveer, the Netherlands) applied to joint, bone or soft tissue in the region of the pain.

#### Quantitative sensory testing

Over the last two decades, QST has been developed to complement traditional neurological bedside examination in the analysis of somatosensory aberrations [19,20]. In theory, greater precision in assessing the functionality of the somatosensory systems is the main advantage of QST over standard bedside examination. QST improves diagnostic procedures and can be helpful for treatment monitoring [11,21]. The protocol we chose is the Nijmegen Aalborg Screening QST Paradigm (NASQ Paradigm) [22]. This maps pain sensitivity at multiple sites by measuring the responses (i.e. painful sensations) evoked by mechanical and electrical non invasive stimuli and measures the patient’s capacity to modulate pain using the Conditioned Pain Modulation [23], previously termed Diffuse Noxious Inhibitory controls (DNIC) or Heterotopic Noxious Conditioning Stimulation (HNCS) [24]. In this study the QST is used to quantify alterations in sensory processing due to the NePC (sensory profiling) in a sub-sample of patients with LBP, NSP and PND (20% of the total population under study, n = 88, equally but randomly divided over all three pain syndromes). Instructions are standardized and will be read to the patient from an instruction sheet. *Pressure Pain Thresholds* (PPT) will be tested by use of an pressure algometer (Somedic sales AB, Hörby, Sweden). PPT will be measured on the left and right bodyside once at each location: Thenar (middle part), musculus trapezius pars median (middle part), musculus rectus femoral (15 cm above patella) and m. abductor hallucis (middle part). *Electrical pain thresholds (EPT)* will be tested by use of the QST-3 device (JNI Biomedical ApS, Klarup, Denmark) on the left and right body side. Measurement locations are the musculus trapezius pars median (middle part) and the musculus rectus femoris (20 cm above patella). *Electrical pain thresholds (EPT)* are assessed and expressed in milli-Ampère. Single pulse evoked pain measurement is performed by one pulse at 150% of the EPT and assessed on a VAS. Summation (i.e. Electric Wind-Up response (E-WUR)) is measured by a train of five pulses at 150% of the EPT and assessed on as VAS. *Conditioned Pain Modulation* (CPM) [23,25] will be assessed. *Electrical Pain Tolerance Thresholds (EPTT)* (test stimulation) are assessed and expressed in milli-Ampère on the m. Rectus femoris contralateral to the dominant hand. The noxious stimulus (conditioning stimulation) is to immerse the dominant hand until the wrist in a bucket filled with water and icecubes (‘Ice water bucket test’) [24] for ‘as long as possible, until the moment that the sensation becomes unbearable and you want to stop directly”. The pain will be recorded every 10 seconds on a NRS. The duration of the immersion (with a maximum of three minutes) will be recorded and the pain at the end of the immersion will be asked. Afterwards, again the EPTT and the PPT on the contra lateral m. rectus femoris are assessed.

#### Douleur neuropathique 4 questions (DN4)

The DN4 [14,15] (© Pfizer bv. Capelle a/d IJssel, the Netherlands) consists of 10 items in total, divided in two questions and two physical examination tests, and is developed to screen components of NeP resulting in a yes/no answer for the presence of NeP. Questions 1 & 2 are sensory descriptors and have to be filled in by the patient or assessed by the physician by interview; questions 3 & 4 are based on a sensory examination by the physician. Question 3 includes two items related to sensory deficits: ‘Is the pain located in an area where the physical examination may reveal one or more of the following characteristics? Touch hypoesthesia and/or pricking hypoesthesia. Question 4 includes 1 item related to evoked pain: ‘In the painful area, can the pain be caused or increased by brushing? Examination of sensitivity to touch (one movement) will be performed with the use of a soft brush (SENSElab^TM^, Brush-05, Somedic AB, Hörby, Sweden). The soft brush will also be used to evaluate tactile (i.e. dynamic mechanical) allodynia (wind-up, with three movements). Examination of sensitivity to touch and pricking will be performed with the use of a Von Frey hair (TOUCH TEST^R^, 5.07, 10.0 g, North Coast Medical Inc., Gilroy, USA). Pressure allodynia (i.e. static mechanical allodynia) is tested by blunt pressure with a finger at a pressure that does not provoke pain in a normally sensitive area [14]. The findings in the physical tests are noted by the physician on the standardized assessment form. The cut-off score for the diagnosis of NeP for the 10-item’ DN4 was determined on 4 times ‘yes’ out of 10 (score range 0-10). This score gave the highest percentage of correctly identified patients (86%), sensitivity (82,9%) and specificity (89,9%). The 7-item’ DN4-interview (score range 0-7) has a cut-off score of 3 times ’yes’ out of 7 which resulted in a percentage of correctly identified patients of 79, 5%, 78% sensitivity and 81,2% specificity [14].

#### PainDetect-Questionnaire (PDQ)

The PDQ (© Pfizer Pharma GmbH 2005, Pfizer bv 2009. Cappelle a/d IJssel, the Netherlands) was developed in Germany [2,16]. The PDQ (© Pfizer Pharma GmbH 2005, Pfizer bv 2009. Cappelle a/d IJssel, the Netherlands) was developed in Germany [2]. The questionnaire can be filled in by the patients themselves and was devised to screen for the presence of a NePC without physical examination. Scoring is performed using a scoring manual and results in a final screening score for the presence of a NePC: ‘negative’, a NePC is unlikely (<15%, score range 0-12); ‘unclear’, result is ambiguous, however a NePC can be present (score range 13-18); or ‘positive’, a NePC is likely (>90%, score range 19-38). The PDQ was tested as a reliable screening tool with a percentage of correctly identified patients of 83% for NeP, sensitivity of 85% and a specificity of 80% [2].

#### Additional questionnaires

*Functioning*: Disability Rating Index (DRI) [26]. The self-administered DRI inquires, in a clinical setting, in 12 items about specified activities (Dressing, Out-door walks, Climbing stairs, Sitting longer time, Standing bent over a sink, Carrying a bag, Making a bed, Running, Light work, Heavy work, Lifting heave objects, Participating in exercise/sports). Score range is from 0 to 100 for each item on a Visual Analogue Scale (VAS). A higher score indicates more disability. The DRI has a good responsiveness (*p* = 0,0001) and a good test-retest correlation of 0,95. The inter- and Intra-rater reproducibility were respectively 0.99 and 0.98 [26]. *Mental health status:* The Hospital Anxiety Depression Scale (HADS) [27] will be used to assess the presence of anxiety and depressive states of patients. This self-administered questionnaire is divided into an anxiety subscale (HADS-A) and a depression subscale (HADS-D), both containing 7-items with a score range of 0-21. The HADS_-Dlv_[28] has a good test-retest reliability for HADS-A, HADS-D and the total HADS (respectively 0.89; 0.86 and 0.91 *p* =0.001). The correlation between the anxiety and the depression subscale was high (0.43 to 0.73) [28]. Based on the review by Bjelland [29] a cut-off score for both the HADS-A and the HADS-D of 8+ gives the best balance in sensitivity and specificity (approximately 0.80 for sensitivity and specificity). *Pain Attribution:* Pain Attribution Scale (PAS) Additional questions to study the attribution of the pain in patients. On a 5-point Likert-scale the patient is asked to rate the influence of several items on the pain complaints. Rating is from ‘totally not of influence on the pain complaints’ to ‘very much of influence on the pain complaints’. *Quality of life: The* RAND 36-item Health Survey (RAND-36) [30] is developed to investigate the health related quality of life. The short, self-administered questionnaire consists of 8 different scales: Physical functioning, social functioning, role limitations (physical problem), role limitations (emotional problem), mental health, pain, general health perception and health change. The psychometric quality of the RAND-36_-Dlv_ was studied by van der Zee [31,32]. *Change (Follow-up, 2 weeks and 3 months):* The Patients Global Impression of Change (PGIC) is a patient rated instrument which measures changes over time on a seven-points scale. Score range is from 1 (very much worse) to 7 (very much improved) [33-35].

### Power calculation

In an unselected cohort of chronic LBP patients, 37% had a high probability of a NePC [2]. Sensitivity and specificity of the PDQ is respectively 85% and 80% [2] and the sensitivity and specificity of the DN4 are respectively 83% and 90% [14]. The expected sensitivity and specificity of the Dutch versions of both questionnaires is set at 80% with an prevalence of 37% and the required lower 95% confidence limit > 0.55. According to Flahault et al the N _*cases*_ is 40. From the equation in the first formula by Flahault, the N_controls_ = 68 [36]. Without prior knowledge of the individual case-control status, the sample size must be determined such that, with high probability (e.g. 95%), the sample contains sufficient numbers of cases and controls. According to the second formula by Flahault et al: N_total_ = 132, in each group. Thus in each group 146 patients will be included (10% drop out). It is expected that this recruitment will be achievable in the 10 general practices, 4 pain treatment centres and 2 departments of neurology chosen.

### Data

All data will be collected from the patients and the physicians on paper and stored by Radboudumc. Data management and monitoring will be performed within MACRO (MACRO, version 4.1.1.3720, Infermed, London, United Kingdom).

### Statistical analysis

To establish the clinimetric quality of the PDQ_-Dlv_ and the DN4_-Dlv_ a comparison will be made between the outcome of both the screening questionnaires and the original pain classification, the Grading system by Treede at al. [1], the bedside examination and the QST measurements. The prevalence of a NePC in patients with LBP and NSP in the Netherlands will be assessed by extrapolating the outcome of this study to the Dutch population. The monitoring and prognosis of the patient over a period of three months by use of the PDQ_-Dlv_ and the DN4_-Dlv_ will be recorded. Data analysis and statistics will be performed by use of Statistical Package for the Social Sciences (SPSS version 18.0, SPSS Inc., Chicago, Illinois, USA). All statistical tests will be two-tailed, for all statistical analysis the type 1 error will be set on 5%.

*Descriptive statistics:* The quantitative variables will be described using mean, standard deviation (SD) and range; Qualitative variables will be described using frequency and percentages. To assess central position, dispersion and distribution of variables, the Kolmogorov-Smirnov test will be used.

*Univariate analysis:* Both the physicians assessments (by rater A and B) will serve as the ‘gold standard’ to assess the presence of a NePC. The internal consistency of both the physicians assessments and the physical examination tests of the DN4 will be separately established for rater A and B by calculating Cronbach’s α that assesses the contribution of each item to the precision of the measurement by both the physicians assessments and the examination items of the DN4 questionnaire.

*Inter-rater reliability:* will be assessed by the agreement of the results obtained by raters A and B for both the physicians assessments and the examination items of the DN4. Agreement was determined by calculating the Cohen’s kappa coefficient.

*Test-retest reliability:* will be assessed for the PDQ and DN4, after two weeks of completion of the questionnaires during the first visit. Stability of the questionnaire will be analyzed by measuring the intra-class correlation coefficient and by use of Cohen’s kappa coefficient of agreement.

*Prognosis and monitoring:* will be assessed for the PDQ and DN4, after three months of completion of the questionnaires during the first visit. Stability of the questionnaire will be analyzed by measuring the intra-class correlation coefficient and by use of Cohen’s kappa coefficient of agreement.

*Correlations:* will be calculated between scores and continuous variables using Pearson correlation coefficient (i.e. correlation between DN4, PDQ and both the physicians assessments). A students-t test for independent groups or a Mann-Whitney’s U test (non-normal distribution) will be used to compare respectively continuous or ordinal variables between patients with and without a neuropatic pain component

*Multivariate analysis:* Sensitivity and specificity percentage of well classified observations and Youden index (i.e. sensitivity + specificity-1) will be calculated for different values of the score of the questionnaire by logistical regression analysis. Positive and negative predictive value for both instruments will also be calculated. The corresponding ROC (receiver operating characteristics) curves will be plotted and AUC calculated using the trapezoid method. Discriminant analysis will be used to analyze complementarily of PDQ and DN4 to each other.

## Discussion

The rationale for this study is to provide detailed information on the clinimetric quality, including test-retest reliability, of the PDQ_-Dlv_ and DN4_-Dlv_ in patients with LBP, NSP or PND regarding of diagnosing a NePC. A validation of these questionnaires is necessary for its use in everyday clinical practice and also in (inter-)national research to make the outcome comparable in different countries. The key question of this study is whether a NePC as assessed by the physician is reflected in the outcome of the PDQ_-Dlv_ and DN4_-Dlv_. In already published articles both questionnaires have proven to be useful in daily clinical practice and for research purposes with good clinimetric qualities [2,14].

This study chose an innovative and wide ranging approach to diagnose a NePC in patients in based on a more reliable identification and qualification of a NePC. In the absence of an internationally accepted ‘gold standard’ [12] the challenge was to find a method to examine the patients in a standardized manner to assess a NePC. The opinion of two physicians about a NePC, the most frequently used standard, will be used in this study and is also used in the original validation studies by Freynhagen et al [2] and Bouhassira et al. [14]. Together with the grading system [1], sensory bed-side examination and QST we will aim to confirm the diagnosis of a NePC, also following the NeuPSIG guidelines for the assessment of neuropathic pain [12]. Screening for nerve damage on basis of sensory bed side examination will be performed by both the physicians. The aim of this examination is to find possible abnormalities suggestive for a relevant lesion or disease which affects the peripheral or central somatosensory system [17]. The value of bed-side examination within the clinical examination is that it will give insight into the pathology and the localization of the lesion or disease which is causing the pain. Touch, pin prick, pressure, cold, heath, vibration and temporal summation were included in the examination to assess the NePC of pain [10,12,18]. For heat allodynia we use a Tip-Therm^R^ in a baby-bottle warmer at 45 degrees Celsius. To our knowledge we are the first to use this method. Because a bottle warmer has a reasonably good thermostat, the temperature of the water inside, and thus the TipTherm^R^, will be kept at the set temperature. In this study we did not use the DFNS sensory testing protocol [19,20] but our own NASQ-protocol. This because we were interested in using QST to assess the altered pain processing, including changes in function of endogenous pain modulation, that may underlie chronic pain conditions, instead of testing small and large nerve-fibre function and the nerve damage related sensory changes [21].

This study will aim to try to define patient groups at risk and to understand and assess the variability and burden of a NePC in individual patients. The PDQ [2] outcome is an ordinal scale, ranging from zero to thirty-eight (a neuropathic pain component is unlikely-neuropathic pain component is likely) and thus the question logically arises whether the PDQ is suitable for the assessment of the amount of nerve damage.

By the choice for a non-selective consecutive patient recruitment in specialized pain clinics, neurology clinics as well as general practices this study aims to validate the PDQ_-Dlv_ and DN4_-Dlv_ in a general, unselected choronic pain population. To date, almost all screening questionnaires are validated in a defined, restricted, population, recruited in specialized pain clinics and pre-selected by precise medical diagnosis (lumbar radicular pain, diabetic polyneuropathy, postherpetic neuralgia etc.). Our choice of a non-selcted population might lead to a lower sensitivity and specificity of the PDQ_-Dlv_ and DN4_-Dlv_ in this study than published in the original validation studies [2,14]. However, the choice for a non-consecutive population has the advantage of providing more information relevant to ordinary clinical practice, in that it is relevant to the unselected ‘general population’.

In conclusion, this study seeks to identify the association between patient’ symptoms, the signs as found in the bedside examination and outcome of the QST measurements, the general and mental health status, functioning, pain attribution and quality of life with regard to the outcome of the PDQ_-Dlv_ and DN4_-Dlv_ in patients with chronic pain due to LBP, NSP or PND.

### Trial status

This study is ongoing. The expected end date of patient recruitment in this study is July 1, 2013.

## Abbreviations

CPM: Conditioned pain modulation; DN4: Douleur neuropathique 4 questions; DN4_-Dlv_: Douleur neuropathique 4 questions dutch language version; DNIC: Diffuse noxious inhibitory control; DRI: Disability rating index; HADS: Hospital anxiety depression scale; LBP: Low back pain; NASQ paradigm: Nijmegen aalborg screening QST paradigm; NeP: Neuropathic pain; NePC: Neuropathic pain component; NRS: Numeric rating scale; NSP: Neck shoulder pain; PAS: Pain attribution scale; PDQ: Pain detect questionnaire; PDQ_-Dlv_: Pain detect questionnaire dutch language version; PGIC: Patients global impression of change; RAND-36: RAND 36-item health survey; Radboudumc: Radboud University medical center; QST: Quantitative sensory testing; VAS: Visual analogue scale.

## Competing interests

The authors declare that they have no competing interests.

## Authors’ contributions

HT, AW, OWS, CvW and KV designed the trial protocol. AW and KV secured funding for the validation study. HT, OWS and KV drafted the manuscript. AW and CvW contributed to the manuscript. All authors read and approved the final manuscript.

## Pre-publication history

The pre-publication history for this paper can be accessed here:

http://www.biomedcentral.com/1471-2377/14/94/prepub

## References

[B1] Treede RD Jensen TS Campbell JN Cruccu G Dostrovsky JO Griffin JW Hansson P Hughes R Nurmikko T Serra J Neuropathic pain: redefinition and a grading system for clinical and research purposes Neurology 2008 70 18 1630 1635 10.1212/01.wnl.0000282763.29778.59 18003941

[B2] Freynhagen R Baron R Gockel U Tolle TR painDETECT: a new screening questionnaire to identify neuropathic components in patients with back pain Curr Med Res Opin 2006 22 10 1911 1920 10.1185/030079906X132488 17022849

[B3] Freynhagen R Baron R The evaluation of neuropathic components in low back pain Curr Pain Headache Rep 2009 13 3 185 190 10.1007/s11916-009-0032-y 19457278

[B4] Pinto RZ Maher CG Ferreira ML Ferreira PH Hancock M Oliveira VC McLachlan AJ Koes B Drugs for relief of pain in patients with sciatica: systematic review and meta-analysis BMJ 2012 344 e497 10.1136/bmj.e497 22331277 PMC3278391

[B5] Attal N Cruccu G Baron R Haanpaa M Hansson P Jensen TS Nurmikko T European Federation of Neurological S: EFNS guidelines on the pharmacological treatment of neuropathic pain: 2010 revision Eur J Neurol 2010 17 9 1113 e1188 10.1111/j.1468-1331.2010.02999.x 20402746

[B6] Dieleman JP Kerklaan J Huygen FJ Bouma PA Sturkenboom MC Incidence rates and treatment of neuropathic pain conditions in the general population Pain 2008 137 3 681 688 10.1016/j.pain.2008.03.002 18439759

[B7] Schers H Bor H van den Hoogen H van Weel C What went and what came? Morbidity trends in general practice from the Netherlands Eur J Gen Pract 2008 14 Suppl 1 13 24 18949639 10.1080/13814780802436051

[B8] Van Den Linden MWWGP De Bakker DH Schellevis FG Tweede nationale studie naar ziekten en verrichtingen in de huisartsenpraktijk: klachten een aandoeningen in de bevolking en in de huisartspraktijk 2004 Utrecht/Bilthoven: NIVEL/RIVM

[B9] Vissers KC The clinical challenge of chronic neuropathic pain Disabil Rehabil 2006 28 6 343 349 10.1080/09638280500287270 16492630

[B10] Baron R Binder A Wasner G Neuropathic pain: diagnosis, pathophysiological mechanisms, and treatment Lancet Neurol 2010 9 8 807 819 10.1016/S1474-4422(10)70143-5 20650402

[B11] Cruccu G Sommer C Anand P Attal N Baron R Garcia-Larrea L Haanpaa M Jensen TS Serra J Treede RD EFNS guidelines on neuropathic pain assessment: revised 2009 Eur J Neurol 2010 17 8 1010 1018 10.1111/j.1468-1331.2010.02969.x 20298428

[B12] Haanpaa M Attal N Backonja M Baron R Bennett M Bouhassira D Cruccu G Hansson P Haythornthwaite JA Iannetti GD Jensen TS Kauppila T Nurmikko TJ Rice AS Rowbotham M Serra J Sommer C Smith BH Treede RD NeuPSIG guidelines on neuropathic pain assessment Pain 2011 152 1 14 27 10.1016/j.pain.2010.07.031 20851519

[B13] Bennett MI Attal N Backonja MM Baron R Bouhassira D Freynhagen R Scholz J Tolle TR Wittchen HU Jensen TS Using screening tools to identify neuropathic pain Pain 2007 127 3 199 203 10.1016/j.pain.2006.10.034 17182186

[B14] Bouhassira D Attal N Alchaar H Boureau F Brochet B Bruxelle J Cunin G Fermanian J Ginies P Grun-Overdyking A Jafari-Schluep H Lantéri-Minet M Laurent B Mick G Serrie A Valade D Vicaut E Comparison of pain syndromes associated with nervous or somatic lesions and development of a new neuropathic pain diagnostic questionnaire (DN4) Pain 2005 114 1–2 29 36 15733628 10.1016/j.pain.2004.12.010

[B15] Van Seventer R Vos C Meerding W Mear I Le Gal M Bouhassira D Huygen FJ Linguistic validation of the DN4 for use in international studies Eur J Pain 2010 14 1 58 63 10.1016/j.ejpain.2009.01.005 19282208

[B16] Timmerman H Wolff AP Schreyer T Outermans J Evers AW Freynhagen R Wilder Smith OH Van Zundert J Vissers KC Cross-Cultural Adaptation to the Dutch Language of the PainDETECT-Questionnaire Pain Pract 2013 13 3 206 214 10.1111/j.1533-2500.2012.00577.x 22776283

[B17] Haanpaa ML Backonja MM Bennett MI Bouhassira D Cruccu G Hansson PT Jensen TS Kauppila T Rice AS Smith BH Treede RD Baron R Assessment of neuropathic pain in primary care Am J Med 2009 122 10 Suppl S13 S21 19801048 10.1016/j.amjmed.2009.04.006

[B18] Cruccu G Truini A Tools for assessing neuropathic pain PLoS Med 2009 6 4 e1000045 10.1371/journal.pmed.1000045 19360134 PMC2661248

[B19] Rolke R Baron R Maier C Tolle TR Treede RD Beyer A Binder A Birbaumer N Birklein F Bötefür IC Braune S Flor H Huge V Klug R Landwehrmeyer GB Magerl W Maihöfner C Rolko C Schaub C Scherens A Sprenger T Valet M Wasserka B Quantitative sensory testing in the German Research Network on Neuropathic Pain (DFNS): standardized protocol and reference values Pain 2006 123 3 231 243 10.1016/j.pain.2006.01.041 16697110

[B20] Rolke R Magerl W Campbell KA Schalber C Caspari S Birklein F Treede RD Quantitative sensory testing: a comprehensive protocol for clinical trials Eur J Pain 2006 10 1 77 88 10.1016/j.ejpain.2005.02.003 16291301

[B21] Krumova EK Geber C Westermann A Maier C Neuropathic pain: is quantitative sensory testing helpful? Curr Diabetes Reports 2012 12 4 393 402 10.1007/s11892-012-0282-7 22623149

[B22] Wilder Smith OH A Paradigm-Shift in Pain Medicine: Implementing a Systematic Approach to Altered Pain Processing in Everyday Clinical Practice Based on Quantitative Sensory Testing 2013 Aalborg, Denmark: Center for Sensory-Motor Interaction (SMI), Department of Health Science and Technology, Aalborg University

[B23] Yarnitsky D Conditioned pain modulation (the diffuse noxious inhibitory control-like effect): its relevance for acute and chronic pain states Curr Opin Anaesthesiol 2010 23 5 611 615 10.1097/ACO.0b013e32833c348b 20543676

[B24] Pud D Granovsky Y Yarnitsky D The methodology of experimentally induced diffuse noxious inhibitory control (DNIC)-like effect in humans Pain 2009 144 1–2 16 19 19359095 10.1016/j.pain.2009.02.015

[B25] Yarnitsky D Arendt-Nielsen L Bouhassira D Edwards RR Fillingim RB Granot M Hansson P Lautenbacher S Marchand S Wilder-Smith O Recommendations on terminology and practice of psychophysical DNIC testing Eur J Pain 2010 14 4 339 10.1016/j.ejpain.2010.02.004 20227310

[B26] Salen BA Spangfort EV Nygren AL Nordemar R The disability rating index: an instrument for the assessment of disability in clinical settings J Clin Epidemiol 1994 47 12 1423 1435 10.1016/0895-4356(94)90086-8 7730851

[B27] Zigmond AS Snaith RP The hospital anxiety and depression scale Acta Psychiatr Scand 1983 67 6 361 370 10.1111/j.1600-0447.1983.tb09716.x 6880820

[B28] Spinhoven P Ormel J Sloekers PP Kempen GI Speckens AE Van Hemert AM A validation study of the Hospital Anxiety and Depression Scale (HADS) in different groups of Dutch subjects Psychol Med 1997 27 2 363 370 10.1017/S0033291796004382 9089829

[B29] Bjelland I Dahl AA Haug TT Neckelmann D The validity of the Hospital Anxiety and Depression Scale. An updated literature review J Psychosom Res 2002 52 2 69 77 10.1016/S0022-3999(01)00296-3 11832252

[B30] Hays RD Sherbourne CD Mazel RM The RAND 36-Item Health Survey 1.0 Health Econ 1993 2 3 217 227 10.1002/hec.4730020305 8275167

[B31] VanderZee KI Sanderman R Heyink J A comparison of two multidimensional measures of health status: the Nottingham Health Profile and the RAND 36-Item Health Survey 1.0 Qual Life Res 1996 5 1 165 174 10.1007/BF00435982 8901380

[B32] VanderZee KI Sanderman R Heyink JW De Haes H Psychometric qualities of the RAND 36-Item Health Survey 1.0: a multidimensional measure of general health status Int J Behav Med 1996 3 2 104 122 10.1207/s15327558ijbm0302_2 16250758

[B33] Collins SL Edwards J Moore RA Smith LA McQuay HJ Seeking a simple measure of analgesia for mega-trials: is a single global assessment good enough? Pain 2001 91 1–2 189 194 11240091 10.1016/s0304-3959(00)00435-8

[B34] Farrar JT Young JP Jr LaMoreaux L Werth JL Poole RM Clinical importance of changes in chronic pain intensity measured on an 11-point numerical pain rating scale Pain 2001 94 2 149 158 10.1016/S0304-3959(01)00349-9 11690728

[B35] Fischer D Stewart AL Bloch DA Lorig K Laurent D Holman H Capturing the patient's view of change as a clinical outcome measure JAMA 1999 282 12 1157 1162 10.1001/jama.282.12.1157 10501119

[B36] Flahault A Cadilhac M Thomas G Sample size calculation should be performed for design accuracy in diagnostic test studies J Clin Epidemiol 2005 58 8 859 862 10.1016/j.jclinepi.2004.12.009 16018921

